# Young age at diagnosis is associated with better prognosis in stage IV breast cancer

**DOI:** 10.18632/aging.102536

**Published:** 2019-12-11

**Authors:** Wei Liu, Xi-feng Xiong, Yu-zhen Mo, Wei-guang Chen, Mei Li, Rong Liang, Zhi-biao Zhang, Zhi Zhang

**Affiliations:** 1 Department of Breast, Guangzhou Red Cross Hospital, Medical College, Jinan University, Guangzhou, Guangdong 510220, China; 2 Guangzhou Institute of Traumatic Surgery, Guangzhou Red Cross Hospital, Medical College, Jinan University, Guangzhou, Guangdong 510220, China; 3 Department of Radiotherapy, Guangzhou Red Cross Hospital, Medical College, Jinan University, Guangzhou, Guangdong 510220, China; 4 Department of Breast, Donghua Hospital of Dongguan, Dongguan, Guangdong 523110, China; 5 Department of Burns and Plastic Surgery, Guangzhou Red Cross Hospital, Medical College, Jinan University, Guangzhou, Guangdong 510220, China

**Keywords:** young age, stage IV breast cancer, prognosis, molecular subtypes

## Abstract

Numerous studies have shown that young age is a risk factor in early breast cancer. But for stage IV breast cancer, it is unclear whether age has a similar effect on patient survival. We collected and analyzed data from patients with stage IV breast cancer between January 2010 and December 2015 in SEER database. Multivariate Cox proportional hazard model was used in this study. 13,069 patients with stage IV breast cancer were included in the analysis, of which 1,135 were young breast cancer patients (≤40 years old). In a multivariate analysis that adjusted for sociodemographic factors, clinical-pathological characteristics and therapeutic methods, the risk of death in patients with stage IV ≤40 years was significantly reduced (hazard ratio [HR], 0.72; 95% CI, 0.65-0.79). Subgroup analyses showed that, with the same adjustment of all factors, young age only significantly reduced the risk of death in patients with luminal A (HR, 0.78; 95% CI, 0.68-0.89) and luminal B (HR 0.46; 95% CI, 0.35-0.60) subtypes. Young age at diagnosis is associated with better survival in patients with stage IV breast cancer. The effect of young age at diagnosis on the survival outcome of stage IV breast cancer varies by subtypes.

## INTRODUCTION

Breast cancer is one of the most common female malignancies worldwide [1]. Young breast cancer is a special group of breast cancer with relatively specific biological behavior and prognosis. Currently, there is no uniform standard for the definition of the age range of young breast cancer. Most literature and studies define young breast cancer patients as those who are newly diagnosed breast cancer at the age of less than or equal to 40. The European Association of Breast Cancer Specialists also chooses the threshold of the age of young breast cancer to be less than or equal to 40 years old. It was reported that breast cancer patients younger than 40 years old account for about 6-7% of the total breast cancer population [2]. Some studies have shown that young breast cancer patients may have a higher proportion of aggressive subtypes, and may have a poor prognosis and a later clinical stage of the tumor [2–5]. A statistical result showed that patients diagnosed younger than 40 years of age had a five-year mortality rate of 85% vs. 90% compared with those older than 40 years. A growing body of research has also shown that younger women are more likely to develop more aggressive breast cancer subtypes that may contain a higher percentage of HER2 overexpressed or triple negative breast cancer [6, 7]. A large number of studies indicate that young age is a risk factor for poor prognosis in early breast cancer, it is unclear whether the prognosis of young women with stage IV breast cancer is worse than that of elderly patients.

The Surveillance, Epidemiology, and End Results (SEER) database is the authoritative cancer statistics database of the United States. The SEER database records information on the morbidity, mortality, and survival outcome of millions of malignant tumor patients in some states and counties in the United States. The SEER database is a population-based cancer registration system that covers 28% of the US population. A large enough sample size allows us to more accurately estimate the effect of age on the survival outcome of stage IV breast cancer at initial diagnosis. The purpose of this study is to determine the effect of age on the survival outcome of stage IV breast cancer and to evaluate the effect of molecular subtypes on age-related differences in survival outcomes.

## RESULTS

From Table 1, the sociodemographic factors, clinical-pathological characteristics, and therapeutic methods of patients classified by age at diagnosis can be seen. In total, 13,069 patients with stage IV breast cancer were included in the analysis, of which 1,135 were ≤40 years old at the time of diagnosis and their average age was 34.77 years. The median follow-up time for the entire cohort was 20 months, ranging from 11.22 months of triple negative subtype to 25.03 months of luminal B subtype. Young stage IV patients included a higher proportion of non-whites, patients receiving Medicaid, unmarried or single patients than those older patients. Younger patients are also had a higher proportion of high histological grades and aggressive subtypes(HER2-enriched and triple negative subtypes). The proportion of luminal B subtype, HER2 subtype and triple negative subtype was higher in patients younger than or equal to 40 years (P < 0.001). Moreover, the proportion of patients receiving chemotherapy and radiotherapy was higher in young age patients (P < 0.001).

**Table 1:** Descriptive characteristics of 13,069 patients with stage IV breast cancer based on age of diagnosis

	**Age at diagnosis(years)**					
	**≤40 (N=1,135)**	**41-50 (N=2,030)**	**51-60 (N=3,464)**	**61-70 (N=3,259)**	**>70 (N=3,181)**	***P***
	**No. (%)**	**No. (%)**	**No. (%)**	**No. (%)**	**No. (%)**	
**Race**					<0.001	
White	735(64.8)	1410(69.5)	2459(71.0)	2513(77.1)	2595(81.6)	
Black	267(23.5)	421(20.7)	661(19.1)	501(15.4)	396(12.4)	
Asian or Pacific Islander	120(10.6)	173(8.5)	309(8.9)	212(6.5)	169(5.3)	
American Indian/Alaska Native	9(0.8)	19(0.9)	26(0.8)	21(0.6)	10(0.3)	
Unknown	4(0.4)	7(0.3)	9(0.3)	12(0.4)	11(0.3)	
**Gender**					0.266	
Female	1127(99.3)	2008(98.9)	3430(99.0)	3213(98.6)	3141(98.9)	
Male	8(0.7)	22(1.1)	64(1.0)	46(1.4)	40(1.3)	
**Insurance status**					<0.001	
Medicaid	345(30.4)	557(27.4)	909(26.2)	549(16.8)	335(13.5)	
Insured	710(62.6)	1319(65.0)	2262(65.3)	2525(77.5)	2753(86.5)	
Uninsured	59(5.2)	119(5.9)	219(6.3)	138(4.2)	73(0.7)	
Unknown	21(1.9)	35(1.7)	74(2.1)	47(1.4	70(2.2)	
**Marital status**					<0.001	
Married	568(50.0)	1034(50.9)	1625(46.9)	1466(45.0)	980(30.8)	
Divorced/ Widowed/ Separated	76(6.7)	324(16.0)	717(20.7)	953(29.2)	1653(52.0)	
Unmarried/Single /Domestic Partner	436(38.4)	587(28.9)	920(26.6)	640(19.6)	348(10.9)	
Unknown	55(4.8)	85(4.2)	202(5.8)	200(6.1)	200(6.3)	
**Histological grades**					<0.001	
I	32(2.8)	96(4.7)	185(5.3)	222(6.8)	250(7.9)	
II	335(29.5)	646(31.8)	1156(33.4)	1107(34.0)	1155(36.3)	
III	634(55.9)	978(48.2)	1549(44.7)	1294(39.7)	1106(34.8)	
Unknown	134(11.8)	310(15.3)	574(16.6)	636(19.5)	670(21.1)	
**Molecular subtypes**					<0.001	
Luminal A	514(45.3)	1106(54.5)	1916(55.3)	2016(62.2)	2121(66.7)	
Luminal B	292(25.7)	391(19.3)	650(18.8)	513(15.7)	422(13.3)	
HER2 enriched	155(13.7)	230(11.3)	389(11.2)	275(8.4)	213(6.7)	
Triple negative	174(15.3)	303(14.9)	509(14.7)	445(13.7)	425(13.4)	
**Chemotherapy**					<0.001	
Yes	940(82.8)	1482(73.0)	2263(65.3)	1805(55.4)	938(29.5)	
No/Unknown	195(17.2)	548(27.0)	1201(34.7)	1454(44.6)	2243(70.5)	
**Radiotherapy**					<0.001	
Yes	450(39.6)	777(38.3)	1253(36.2)	1064(32.6)	815(25.6)	
No/Unknown	685(60.4)	1253(61.7)	2211(63.8)	2195(67.4)	2366(74.4	
**Surgery**					0.006	
Yes	361(31.8)	636(31.3)	1136(32.8)	1000(30.7)	910(28.6)	
No/Unknown	774(68.2)	1394(68.7)	2328(67.2)	2259(69.3)	2271(71.4)	
**ER status**					<0.001	
Positive	789(69.5)	1451(71.5)	2495(72.0)	2480(76.1)	2499(78.6)	
Negative	345(30.4)	574(28.3)	969(28.0)	777(23.8)	677(21.3)	
Borderline/Unknown	1(0.1)	4(0.2)	0(0)	2(0.1)	5(0.1)	
**PR status**					<0.001	
Positive	666(58.7)	1217(60.0)	1921(55.5)	1988(61.0)	1973(62.0)	
Negative	465(41.0)	798(39.3)	1521(43.9)	1253(38.4)	1181(37.1)	
Borderline/Unknown	4(0.4)	15(0.7)	22(0.7)	18(0.5)	27(0.9)	
**HER2 status**					<0.001	
Positive	447(39.4)	621(30.6)	1039(30.0)	788(24.2)	635(20.0)	
Negative	688(60.6)	1409(69.4)	2425(70.0)	2471(75.8)	2546(80.)	

ER: estrogen receptor; PR: progesterone receptor; HER2: human epidermal growth factor receptor 2.

### Correlation between age and breast cancer mortality

**Figure 1:**
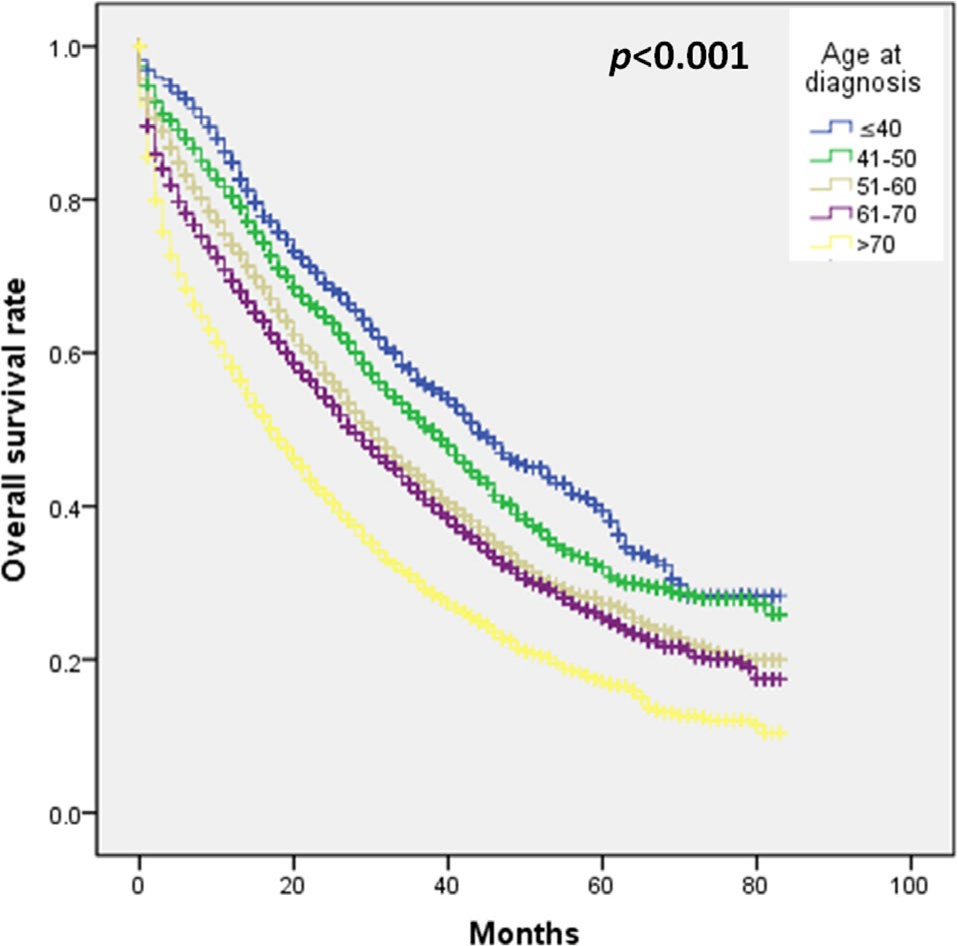
**The effect of age at diagnosis on survival outcome in patients with stage IV breast cancer.**

Firstly, we performed a survival analysis and found that the age of diagnosis≤40 years predicted a better survival outcome in patients with stage IV breast cancer (Figure 1). In Table 2, after adjusting for sociodemographic factors, multivariate analysis showed that patients younger than or equal to 40 years of age had a 30% lower risk of death than patients aged 51 to 60 years (HR, 0.70; 95% CI, 0.63–0.77). After additional adjustments in molecular subtypes and histological grades, the results showed that patients who were younger than or equal to 40 years of age still had a 34% lower risk of death than those aged 51 to 60 (HR, 0.66; 95 %CI, 0.60–0.73). After further adjustment of therapeutic methods (chemotherapy, surgery, radiotherapy), the impact still existed, and patients younger than or equal to 40 years of age had a 28% lower risk of death than those aged 41-50 years(HR, 0.66; 95 %CI, 0.60–0.73). In the model of adjusted for all factors, compared with patients aged 51 to 60 years, the risk of death in patients aged 41 to 50 years (HR, 0.85; 95% CI, 0.79–0.92) was also reduced by 15%, but age >70-year-old patients have a 38% increase in the likelihood of death (HR, 1.38; 95% CI, 1.30–1.47). Under the conditions of adjusting for all factors, 61 to 70 years old patients (HR, 0.8; 95% CI, 0.70–1.00) also had no difference in survival rate compared with patients aged 51 to 60 years. The results of breast cancer-specific survival analysis further demonstrate that younger age is associated with better prognosis in stage IV breast cancer (Supplementary Table 1).

**Table 2:** Univariate and multivariate analysis of breast cancer survival outcomes based on age at diagnosis.

**Age at diagnosis (years)**	**No. of patients**	**Breast cancer deaths, No. (%)**	**HR (95%CI)**	HR (95%CI) ^*^	**HR (95%CI) ^&^**	**HR (95%CI) ^#^**
**≤40**	1135	544(47.9)	0.70(0.63-0.77)	0.69(0.62-0.75)	0.66(0.60-0.73)	0.72(0.65-0.79)
**41-50**	2030	1078(53.1)	0.82(0.76-0.88)	0.83(0.77-0.89)	0.82(0.76-0.88)	0.85(0.79-0.92)
**51-60**	3464	2037(58.8)	Reference	Reference	Reference	Reference
**61-70**	3259	1989(61.0)	1.09(1.03-1.16)	1.10(1.04-1.17)	1.14(1.07-1.21)	1.09(1.03-1.16)
**>70**	3181	2252(70.8)	1.53(1.44-1.63)	1.55(1.46-1.65)	1.64(1.54-1.74)	1.38(1.30-1.47)

**HR**: hazard ratio; **CI**: confidence interval.

*Adjusted for race, insurance status, marital status.

^**&**^Adjusted for race, insurance status, marital status, molecular subtype, histological grade.

**^#^** Adjusted for race, insurance status, marital status, molecular subtype, histological grade, therapeutic methods (Surgery [yes/no], chemotherapy [yes/no], radiotherapy [yes/no]).

### Correlation between age and prognosis based on tumor subtype stratification

We analyzed the relationship between different age groups and the prognosis of breast cancer patients after stratification by molecular subtypes (Table 3). In stage IV breast cancer patients with luminal A subtype (n=7,683), after adjusting for sociodemographic factors, clinical-pathological characteristics and therapeutic methods, the risk of death was significantly lower in patients younger than or equal to 40 years compared with patients aged 51 to 60 years (HR, 0.72; 95% CI, 0.65-0.79). In addition, young age patients also had a lower death risk (HR, 0.46; 95% CI, 0.35-0.60) in stage IV breast cancer patients with luminal B subtype. For HER2-enriched subtype stage IV breast cancer patients, we also observed that younger patients had a better prognosis than patients aged 51 to 60 years (HR, 0.70; 95% CI, 0.52-0.94) after controlling sociodemographic factors and clinical-pathological characteristics. However, in HER2-enriched subtype stage IV breast cancer patients, after adjusted for sociodemographic factors, clinical-pathological characteristics and therapeutic methods, there was no significant difference in prognosis between younger than or equal to 40 years and of age 51 to 60 years (HR, 0.77; 95% CI, 0.58-1.04). For patients with triple-negative subtype stage IV breast cancer, after controlling different methods, we observed no significant difference in the prognosis between patients aged younger than or equal to 40 years and those aged 51 to 60 years (HR, 0.92; 95% CI, 0.76-1.11).

**Table 3:** Multivariate analysis of age and breast cancer survival outcomes based on molecular subtypes.

**Subtypes and Age(years)**	**No. of breast cancers**	**Breast cancer deaths, No. (%)**	HR (95%CI) ^*^	**HR (95%CI) ^&^**	**HR (95%CI) ^#^**
**Luminal A**					
**≤40**	514	262(51.0)	0.76(0.66-0.87)	0.75(0.66-0.86)	0.78(0.68-0.89)
**41-50**	1106	563(50.9)	0.79(0.71-0.87)	0.79(0.71-0.87)	1.03(0.72-0.89)
**51-60**	1916	1110(57.9)	Reference	Reference	Reference
**61-70**	2026	1161(57.3)	1.04(0.95-1.12)	1.03(0.95-1.12)	1.01(0.93-1.10)
**>70**	2121	1440(67.9)	1.49(1.37-1.61)	1.49(1.38-1.61)	1.39(1.28-1.51)
**Luminal B**					
**≤40**	292	71(24.3)	0.40(0.31-0.51)	0.40(0.31-0.51)	0.46(0.35-0.60)
**41-50**	291	153(39.1)	0.74(0.61-0.90)	0.74(0.61-0.90)	0.78(0.65-0.95)
**51-60**	650	320(49.2)	Reference	Reference	Reference
**61-70**	513	280(54.6)	1.21(1.03-1.43)	122(1.04-1.43)	1.19(1.01-1.40)
**>70**	422	283(67.1)	1.91(1.53-2.24)	1.92(1.63-2.25)	1.48(1.25-1.74)
**HER2 enriched**					
**≤40**	155	59(38.1)	0.71(0.53-0.95)	0.70(0.52-0.94)	0.77(0.58-1.04)
**41-50**	230	112(48.7)	0.96(0.76-1.22)	0.95(0.75-1.20)	1.04(0.82-1.31)
**51-60**	389	186(47.8)	Reference	Reference	Reference
**61-70**	275	163(59.3)	1.34(1.08-1.65)	1.31(1.06-1.62)	1.35(1.09-1.67)
**>70**	213	165(77.5)	2.42(1.96-2.99)	2.36(1.91-2.92)	1.75(1.40-2.18)
**Triple Negative**					
**≤40**	174	142(81.6)	0.88(0.72-1.06)	0.87(0.72-1.05)	0.92(0.76-1.11)
**41-50**	303	250(82.5)	0.90(0.77-1.05)	0.90(0.77-1.05)	0.94(0.80-1.10)
**51-60**	509	421(82.7)	Reference	Reference	Reference
**61-70**	445	385(86.5)	1.19(1.04-1.37)	1.19(1.03-1.04)	1.10(0.96-1.26)
**>70**	425	364(85.6)	1.32(1.15-1.52)	1.31(1.14-1.51)	1.04(0.90-1.20)

HER2: human epidermal growth factor receptor 2; HR: hazard ratio; CI: confidence interval.

^*^Adjusted for race, insurance status, marital status.

^**&**^Adjusted for race, insurance status, marital status, molecular subtype, histological grade.

**^#^** Adjusted for race, insurance status, marital status, molecular subtype, histological grade, therapeutic methods (Surgery [yes/no], chemotherapy [yes/no], radiotherapy [yes/no]).

After adjusting for sociodemographic factors, clinicopathological features, and treatments, the risk of death was significantly lower in ≤40 patients compared with patients aged 51-60 years in stage IV breast cancer of white race (HR, 0.70; 95% CI, 0.62-0.79) or black race (HR, 0.72; 95% CI, 0.60-0.87). For Asian or Pacific Islander(HR, 0.75; 95% CI, 0.55-1.02), American Indian/Alaska Native patients(HR, 0.28; 95% CI, 0.06-1.25), patients ≤40 years of age also showed better prognosis than patients aged 51-60 years, although there was no statistical difference due to the small sample size (Table 4).

**Table 4:** Multivariate analysis of age and breast cancer survival outcomes based on Race/ethnicity^*^.

**Age at diagnosis (years)**	**No. of patients**	**Breast cancer deaths, No. (%)**	**HR(95%CI)**				
			**White (N=9,712)**	**Black (N=2246)**	**Asian or Pacific Islander (N=983)**	**American Indian /Alaska Native (N=85)**	**Chinese (N=126)**
≤40	1131	534	0.70(0.62-0.79)	0.72(0.60-0.87)	0.75(0.55-1.02)	0.28(0.06-1.25)	0.58(0.06-1.25)
41-50	2023	1076	0.83(0.76-0.91)	0.90(0.77-1.04)	0.85(0.65-1.10)	0.59(0.26-1.34)	0.82(0.26-1.34)
51-60	3455	2036	Reference	Reference	Reference	Reference	Reference
61-70	3247	1985	1.13(1.05-1.22)	1.06(0.92-1.22)	0.91(0.72-1.17)	1.50(0.59-1.71)	1.50(0.59-1.71)
>70	3170	2250	1.45(1.35-1.56)	1.24(1.07-1.44)	1.26(0.98-1.62)	1.99(1.01-3.84)	1.99(1.01-3.84)

^*^Adjusted for insurance status, marital status, molecular subtype, histological grade, therapeutic methods (Surgery [yes/no], chemotherapy [yes/no], radiotherapy [yes/no]).

## DISCUSSION

A large number of studies have found that young breast cancer have a worse prognosis than older breast cancer, because young breast cancer contain a higher proportion of aggressive molecular subtypes, such as triple negative and HER-2 positive subtypes [5, 6, 8, 9]. Numerous studies have shown that young age is a risk factor for poor prognosis in early breast cancer. But for stage IV breast cancer, it is unclear whether young age has a similar effect on patient survival. Here, through a large sample, population-based study we have found that age still an independent prognostic factor for stage IV breast cancer, but young age predicted better prognosis for stage IV breast cancer, not worse. At the same time, we conduct a stratified analysis according to molecular subtypes and found that the effect of young age on the prognosis of stage IV breast cancer patients seems to vary with molecular subtypes. For patients with luminal subtypes breast cancer, young age appears to be a protective prognostic factor.

According to reports, young age is an independent influencing factor for poor survival outcome in early breast cancer. But surprisingly, we observed that young age predicting a better prognosis for stage IV breast cancer in this study. Purushotham et al. found that although the risk of distant metastasis decreased with age, the risk of death was increased in patients with bone metastases >50 years of age and visceral metastases >70 years of age when metastatic diagnosis [10]. The results of Purushotham et al. are consistent with the findings of our research to some extent. The possible reasons for the better prognosis of young age-related stage IV breast cancer found in this study are as follows. First, young breast cancer patients are more likely to receive standardized treatments. For example, in this study, we also found that patients ≤ 40 years of age received a higher percentage of chemotherapy and radiotherapy. In addition, as age increases, the likelihood of patients with underlying diseases such as hypertension and diabetes increases, making it more difficult to use invasive systemic therapy in elderly patients with stage IV breast cancer. Third, tumor biology may play an important role, such as the weakened immune system in the elderly. Previous studies have shown that with human aging, the immune system is gradually declining, and a series of changes occur in the innate and adaptive immune system [11]. In addition, the adaptive immune system may also be severely damaged with age. With aging, T and B cell functions may deteriorate and have some degree of defects [12, 13]. Moreover, the B cell lineage diversity of the elderly may also drop dramatically [14]. Finally, the impact of factors such as medical insurance or medical assistance cannot be ignored. Medicaid is a US government program that provides medical support to some low-income individuals and families. As a health care program, Medicaid is designed to help people who are unable to pay for health care to enjoy basic health care. It pays for health care providers directly for the recipients, who only pay a small fee for certain medical services. Because the cost of treatment for metastatic breast cancer is often very high, especially some new drugs, such as targeted drugs, CDK4/6 inhibitors, PD1/PD-L1 inhibitors, etc.

With the in-depth study of the biological behavior of breast cancer, the effect of young age on the prognosis of breast cancer patients is considered to be closely related to molecular subtypes and highly subtype dependent. Partridge et al. [4] studied 17,575 patients with stage I to III breast cancer and found that young age at diagnosis only significantly increased the risk of death in patients with luminal A subtype. Our study also further confirmed that the effect of age on the prognosis of breast cancer patients is also highly subtype dependent. Stratification analysis in this study showed that, with the adjustment of all factors, age ≤40 years only significantly lowered risk of death in patients with luminal A (HR, 0.78; 95% CI, 0.68 -0.89) and luminal B subtypes (HR 0.46; 95% CI, 0.35-0.60), but did not decrease that of HER2-enriched (HR, 0.77; 95% CI, 0.58-1.04) and triple-negative subtypes (HR, 0.92; 95% CI, 0.76-1.11). Sheridan et al. [15] and Anders et al. [16] found that the risk of recurrence was similar in young women and older women after adjusting for other prognostic factors for triple-negative breast cancer. A retrospective analysis of a large randomized controlled trial found that for HER2-positive breast cancer patients, young age was not associated with early recurrence risk [17].

To the best of our knowledge, this study is currently the largest comprehensive study to assess the impact of age on the prognosis of stage IV breast cancer, and has adjusted for sociodemographic factors, clinical-pathological characteristics and therapeutic methods. However, there are some potential limitations in this study, which should be considered when we interpret the results. First, this study did not adjust for the effects of endocrine therapy and anti-HER2 therapy, which have important impacts for the prognosis of ER and/or PR positive and HRE2-positive breast cancer. Secondly, the conclusions of this study are mainly based on the study of stage IV breast cancer. It is necessary to further study whether young age has similar effects on recurrent metastatic breast cancer. After all, there is a certain degree of difference in the performance and survival rate between stage IV and recurrent metastatic breast cancer [18–20].

## CONCLUSIONS

This study demonstrates that young age is a favorable prognostic factor for stage IV breast cancer. At the same time, the impact of young age on the prognosis of stage IV breast cancer is also highly subtype dependent. Young age seems to be associated with better survival outcome only in luminal subtypes stage IV breast cancer, but not in HER-2 enriched and triple-negative stage IV breast cancer.

## MATERIALS AND METHODS

### Patients

We collected data from the Surveillance, Epidemiology, and End Results (SEER) database for newly diagnosed stage IV (American Joint Committee on Cancer [AJCC] Cancer Staging, 7^th^ Edition) adult invasive breast cancer between January 1^st^, 2010 and December 31^st^, 2015. Patients with unknown age or other malignancies were excluded. Subsequently, we excluded patients who were diagnosed by autopsy or death certificate, as well as patients with no follow-up records or molecular subtypes unclear. This study was approved by the review board of the author's institution.

To examine the impact of diagnosis age on prognosis, we stratified every 10 years between 40 and 70 years old: ≤40, 41-50, 51-60, 61-70, and > 70 years of age. We used the 51 to 60 age group as a comparative reference, because it has the largest number of cases and allows us to compare the differences between young age and old age breast cancer.

For breast cancer subtypes, we divided patients into four subtypes according to the SEER database: Luminal A subtype (ER-positive and/or PR-positive, HER2-negative), Luminal B subtype (ER-positive and/or PR-positive, HER2-positive), HER2- enriched subtype (ER-negative, PR-negative and HER2-positive) and triple-negative subtype (ER-negative, PR-negative and HER2-negative). Based on race recoding, we divided the race into four groups: White (W), Black (B), American Indian/Alaska Native (AI), and Asian or Pacific Islander (API).

### Statistical analysis

Chi-square test is used to examine differences in clinical-pathological characteristics between different age groups. Univariate and multivariate Cox proportional hazard regression was used to assess hazard ratios (HRs) and 95% CI for stage IV breast cancer deaths. Kaplan-Meier survival analysis was used to assess survival differences between different age groups.

### Ethics approval

The study was approved by the Ethics Committee of Guangzhou Red Cross Hospital.

## Supplementary Material

Supplementary Table 1
