# Supplementary material for: Young age at diagnosis is associated with better prognosis in stage IV breast cancer
Source: Aging (Albany NY). 2019 Dec 11;11(23):11382–90. doi: 10.18632/aging.102536 (PMC6932875; doi:10.18632/aging.102536)
Supplement: Supplementary Table 1 [file aging-11-102536-s001..pdf]

## SUPPLEMENTARY TABLE

**Supplementary Table 1. Multivariate analysis of age and breast cancer special survival by competitive risk model.**

| Age at diagnosis (years) | No. of patients | Total deaths, No. (%) | Deaths due to this cancer | HR (95%CI) * |
|---|---|---|---|---|
| **≤40** | 1135 | 544 (47.9) | 505 (44.5) | 0.73 (0.67–0.81) |
| **41–50** | 2030 | 1078 (53.1) | 1019 (50.2) | 0.86 (0.80–0.92) |
| **51–60** | 3464 | 2037 (58.8) | 1919 (55.4) | Reference |
| **61–70** | 3259 | 1989 (61.0) | 1814 (55.7) | 1.04 (0.98–1.11) |
| **>70** | 3181 | 2252 (70.8) | 1962 (62.70) | 1.19 (1.11–1.28) |
